# Evaluation of the COBAS Core II immunochemistry analyser

**DOI:** 10.1155/S1463924696000259

**Published:** 1996

**Authors:** Jochem Kötting, Gabriele Heinemann, Isolde Salb, Herbert Maier-Lenz

**Affiliations:** Tumor Biology Center (Klinik für Tumorbiologie) Institute for Clinical Pharmacology Central Laboratory Freiburg im Breisgau D-79106 Germany


Journal of Automatic Chemistry, Vol. 18, No. 6 (November–December 1996) pp. 205–215

### Evaluation of the COBAS Core II immunochemistry analyser

**Jochem Kötting, Gabriele Heinemann, Isolde Salb and Herbert Maier-Lenz**

*Tumor Biology Center (Klinik für Tumorbiologie), Institute for Clinical Pharmacology, Central Laboratory, D-79106 Freiburg im Breisgau, Germany*

*A continuous random access immunoassay analyser, the COBAS Core II, was evaluated in the central laboratory of the Tumor Biology Center in Freiburg, Germany for three months. The performance of six COBAS Core immunoassays (AFP, CA 15-3, CEA, PSA, TSH and FT4) were investigated. Intra-assay imprecision (CVs ranging from 1·8 to 4·9%) and inter-assay imprecision (CVs ranging from 2% to 8·5%) was good. Accuracy was verified with three commercially available quality control sera. In particular, the automatic dilution of the COBAS Core II proved useful and gave superior reproducibility compared to manual dilution. A method comparison with the Abbott AxSYM™ was also performed—using more than 600 routine patient samples. Good correlation was found for all assays (slopes ranging from 0·92–1·28, correlation $r_s = 0{\cdot}93$–$0{\cdot}98$), except for PSA because human specimens were used. The COBAS Core II was evaluated for ease of use and efficiency compared to the Abbott AxSYM. The performance of the COBAS Core II immunoassay analyser was similar to the laboratory's routine analyser; the COBAS Core II required much less operator time. The COBAS Core II was found to be well suited to an oncology laboratory.*

### Introduction

Clinical laboratories are under constant pressure to provide a high-quality service, improve efficiency and keep costs to a minimum so they need diagnostic systems with the highest standards of performance, convenience, and cost effectiveness.

Roche Diagnostics recently launched the COBAS Core II, which is a fully automated, continuous random access immunoassay analyser, capable of running up to 150 tests/hour with a maximum of 18 on board reagents, continuous reloading of samples and reagents and auto-dilution of samples. The COBAS Core II was evaluated in the central laboratory of the Institute for Clinical Pharmacology in the Tumor Biology Center, Freiburg, Germany from July to November 1995. The objectives of the evaluation were to check the analytical performance with selected thyroid and tumour marker parameters, to look at the reliability, ease of use and efficiency of the analyser under routine conditions and in comparison to an analyser already installed in the laboratory.

### Materials and methods

### *COBAS Core II immunoassay analyser*

The COBAS Core II immunoassay analyser (Roche Diagnostics, Switzerland) is a random access automated immunochemistry analyser. The test instrument's menu included 45 tests in a ready-to-use format, with piercable caps comprising assays for oncology, retrovirus, hepatitis, TORCH, thyroid, and fertility testing.

The following assays were performed in this study: COBAS Core EIAs: thyrotropin (TSH), free thyroxine (FT4), thyroxine (T4), triiodothyronine (T3), alpha-1-fetoprotein (AFP), β-human chorionic gonadotropin (β-hCG), cancer antigen 15-3 (CA 15-3), carbohydrate antigen 19-9 (CA 19-9), cancer antigen 125 (CA 125), and prostate specific antigen (PSA), carcinoembryonic antigen (CEA), and the improved version CEA (update). The test principles are described in more detail in the reagents section of this paper.

In the COBAS Core II analyser, samples are analysed in a patient-oriented mode, and STAT tests can be added without interrupting any tests in progress. The instrument offers continuous loading of samples and reagents, supported by LED indicators on the rank platform. Different types of primary tubes, and a variety of secondary tubes, can be used. The COBAS Core II offers automatic pre- and post-dilution of samples with operator definable dilution factors of up to $1{:}10^6$. Barcoded samples, as well as sample and reagent racks, are identified by a laser barcode scanner. Menu-structured software in a Windows NT environment includes an on-line help facility guiding the user through all functions and operations. The COBAS Core II can be linked to a computer.

Most of the COBAS Core assays have a total assay time of less than 60 minutes. The maximum throughput is 150 samples per hour.

The specifications according to IFCC recommendations [1] of the COBAS Core II are shown in table 1.

### *Reagents*

The COBAS Core immunoassays are solid phase enzyme immunoassays (EIA) using competitive binding, sandwich, and antibody detection; test formats are shown in table 2(*a*). Essentially, a large bead, coated with defined amounts of antigen or antibody, is incubated with serum or plasma containing the analyte. Depending on the test format, a washing step may be included before a horseradish peroxidase (HRP)-conjugate is added to detect bound antibody or antigen. This is followed by a final washing step. The substrate $H_2O_2$/3,3′,5,5′-tetramethylbenzidine (TMB) is added to detect the bound HRP-conjugate and the OD is read off at 650 nm. All steps are fully automated on the COBAS Core II immunoassay analyser.

Full calibration or recalibration using one calibrator is possible. In the course of this evaluation, some tests were

*Table 1. Specifications of the COBAS Core II immunochemistry system according to the TFCC guidelines [1].*

| | |
|---|---|
| General information | COBAS CORE II immunochemistry system (Roche Diagnostics, a Division of F. Hoffmann-La Roche Ltd, Grenzacherstrasse 124, CH-4070 Basel).<br>Listing of specification as of October 1995. |
| *Type*: | Random access, sample selective analyser |
| *Method of analysis*: | Heterogeneous enzyme immunoassays utilizing big bead technology as solid phase, horse-radish peroxidase as marker enzyme (conjugate) and 3,3′,5,5′-tetramethyl-benzidine (TMB) as substrate. |
| *Throughput*: | Up to 150 tests per hour, depending on the test mix. |
| *System operation*: | Samples are analysed in patient-oriented mode. STAT tests can be added without interrupting other tests already in progress. Continuous loading of both samples and reagents is possible. |
| *Mode of sampling*: | X, Y, Z robotic transfer mechanism containing the sample and reagent dispenser and the bead dispenser. |
| *Transport*: | Rack system: sample racks and reagent racks loaded on a rack platform with 14 slots.<br>A sample rack needs one slot, a reagent rack needs two slots. Up to 12 sample racks with maximum of 180 samples or up to six reagent racks with a maximum of 18 tests can be loaded at any one time. Each sample rack can accommodate 15 sample tubes, and each reagent rack up to three tests. A red/yellow/green LED display indicates the rack status. |
| Samples | |
| *Nature of specimen*: | Serum, plasma, urine, amniotic fluid. |
| *Specimen pretreatment*: | No specimen pretreatment is required. Automatic pre- and post-dilution with operator definable dilution factors in a range of 1:1–1:1,000,000. |
| *Specimen container*: | Sample tubes with volumes of 3·5 ml up to 10 ml. Secondary cups such as Biocups (700 µl) and Microcups (1500 µl, for example Eppendorf) can be positioned with an adapter. |
| *Specimen identification system*: | Specimen identified by rack- and position barcode. Positive patient identification by barcode possible. The barcode scanner supports the following symbologies: Code 128, Code 3 of 9, Codabar, 2 of 5 Interleaved with up to 20 characters. |
| *Specimen aspiration and dispensing*: | 500 µl syringe driven by linear stepper motor. Sample volume: 3–250 µl selectable in steps of 1 µl. Capacitive level detection is used for level sensing of samples and reagent. Sample carry-over: less than 0·5 ppm checked with HBsAg test. The sample and reagent probes are cleaned after each work cycle. |
| Chemical processor | Discrete. |
| *Test menu*: | Full test menu with 45 assays from the following test groups: oncology, thyroid, fertility, TORCH, hepatitis, retroviruses. |
| *Measurement*: | Spectrophotometric (bichromatic) measurement. Calculation of rate values corresponds to an endpoint measurement against blank. |
| *Calibration*: | Full standards with up to six calibrators (test dependent), recalibration with one or two calibrators possible. Linear interpolation for quantitative tests. |
| *Reagent dispending*: | One 500 µl syringe for reagent, conjugate and diluent and one 1000 µl syringe for substrate, both driven by linear stepper motor.<br>Conjugate/diluent volume: 3–470 µl selectable in steps of 1 µl.<br>Reagent volume: 10–470 µl selectable in steps of 1 µl.<br>Substrate volume: 500 µl (fixed). |
| *Reaction vessel*: | Polystyrene reaction tubes, one tube is used for each test. Tubes are supplied in prepacked cassettes holding 120 tubes. Four tube cassettes can be loaded onto the tube platform. The tubes are automatically transferred from the tube platform to the analyser and back by an X, Y, Z robotic tube transfer mechanism. |
| *Mixing*: | Non-invasive mixing of sample, reagent and conjugate during incubation by intermittent shaking of the incubator at 720–800 rpm, circular shaking mode. Mixing of sample and sample diluent by circular shaking of the workstation A1/2. |
| *Washing*: | Bound/free separation by washing the beads with deionized water. The number of washing steps (one or two) is test dependent. |
| *Incubator and temperature control*: | Thee incubator is automatically loaded and unloaded with up to 297 reaction tubes. Heating by thermistor controlled temperature system, the temperature is maintained at 37°C ± 1°C (air bath). Minimum 5 min needed to reach operating temperature after power on. The incubation time is test dependent and ranges from 15 min (for example oncology assays) to 2 h (retroviruses). |
| Signal measuring: | Spectrophotometer with halogen lamp (spectral range 400–750 nm) and two band pass filters selecting two wavelengths: 650 nm (measuring wave length) and 492 nm (reference wavelength); inaccuracy of wavelength: ±3 nm; Band width: 10 nm ± 3 nm; silicon photodiode used for detection. Linearity range: Abs = 0–2 A with linearity error of < 2%. Measuring range: Abs = 0–3·5 A. Photometric reproducibility: (1 SD) ≤0·002 at 1·0 A, ≤0·01 at 2·0 A. |
| Data handling: | Windows NT operating system with COBAS CORE II user interface including the following features: on-line help facility; Profile and conditional profile testing; automatic or manual result acceptance; absorbance values available for inspection by patient ID or test type; completely integrated system checks with self-diagnostic messages. |

| | | |
|---|---|---|
| *Interfaces*: | Three RS232 bidirectional ports, one unidirectional parallel port for printer LAN for various protocols (optional) | |
| *Processor*: | Intel DX 486 CPU, 16 Mbyte RAM. | |
| *Output*: | 24·1 cm (9½ in.) VGA colour monitor, result output via printer and/or interface. | |
| *Data storage*: | 2·5 in, 350 megabyte hard disk drive, 3·5 in, 1·44 megabyte floppy disk drive. | |
| Requirements | | |
| *Power requirements* | Line voltages: | 100 to 125 V/200 to 240 V. |
| | Current: | 5 A (220 V) or 8 A (110 V). |
| | Line frequency: | 50–60 Hz (±2 Hz). |
| | Power consumption: | 1000 VA maximum. |
| *Power failure*: | Software is protected against momentary power failure for 20 ms by battery. | |
| *Water requirements*: | Laboratory reagent grade type I or double-distilled water < 1·5 μS conductivity. Supply via water reservoir or via direct connection. Water consumption 5 to 7 l per hour in operating mode. | |
| *Waste Handling* | Liquid waste disposal via waste reservoir or via direct connection to sanitary sewer system. | |
| *Environmental conditions*: | Operating temperature: 15–32°C (59–89°F). Relative humidity: maximum 80% at 32°C (89°F). | |
| *Physical Dimensions*: | Weight: 110 kg (242 lb.). Width: 99 cm (39 in.). Depth: 66 cm (26 in.). Height: 76 cm (30 in.). | |

run with full calibration, some with recalibration. All analytes determined with the COBAS Core II were calibrated at the beginning of the evaluation and were recalibrated according to the recommended time interval of minimum 14 days.

### *Abbott AxSYM and Abbott IMx System*

The Abbott AxSYM System (Abbott Laboratories, USA) is an automated random access immunoassay analyser which uses three different technologies: microparticle capture enzyme immunoassay (MEIA); fluores-

*Table 2(a). Specifications of the COBAS EIA assays used with the COBAS CORE II during the evaluation.*

| Analyte[1] | Type of EIA (quantitative) | Measuring range of test | Reference range | Sample volume | Sample type | Incubation time | High dose hook effect |
|---|---|---|---|---|---|---|---|
| AFP | One-step sandwich | 0·25–275 ng/ml | < 7·88 ng/ml (97·5%)[2] | 50 μl | Serum, amniotic fluid | 30 min | > 4·87 · 10⁴ ng/ml |
| β-HCG | One-step sandwich | 1·25–200 mIU/ml | < 4·0 mIU/ml (99·0%)[2] | 50 μl | Serum, plasma | 45 min | > 6 · 10⁶ mIU/ml |
| CA 19-9 | Two-step sandwich | 2–400 U/ml | < 37 U/ml (97·5%)[3] | 50 μl | Serum, plasma | 75 min | NA |
| CA 15-3 | Two-step sandwich | 2–400 U/ml | < 30 U/ml (98%)[3] | 5 μl | Serum, plasma | 75 min | NA |
| CA 125 | One-step sandwich | 0·45–540 U/ml | < 35 U/ml (97·5%)[3] | 50 μl | Serum, heparin-plasma | 45 min | > 2·8 · 10⁵ U/ml |
| CEA | One-step sandwich | 0·2–50 ng/ml[4] | < 5 ng/ml (97·5%)[3] | 50 μl | Serum, plasma | 45 min | > 7 · 10⁵ ng/ml |
| PSA | One-step sandwich | 0·2–120 ng/ml | < 4·0 ng/ml (99%)[3] | 50 μl | Serum, plasma | 30 min | > 2 · 10⁴ ng/ml |
| TSH | One-step sandwich | 0·03–500 mIU/l | 0·2–3·2mIU/l | 100 μl | Serum, plasma | 45 min | > 3 · 000 mIU/l |
| FT4 | Two-step back titration | 2·3–110 pmol/l | 10–27 pmol/l | 5 μl | Serum, plasma | 47 min | NA |
| T3 | One-step competitive | 0·15–7·0 ng/ml | 0·8–2·0 ng/ml | 50 μl | Serum, EDTA, heparin-plasma | 45 min | NA |
| T4 | One-step competitive | 0·35–33·0 μg/dl | 5·0–11·3 μg/dl | 20 μl | Serum, heparin-plasma | 45 min | NA |

[1] AFP = alpha-1-fetoprotein; β-HCG = β-human chorionic gonadotropin; CA 19-9 = carbohydrate antigen 19-9; Ca 15-3 = cancer antigen 15-3; Ca 125 = cancer antigen 125; CEA = carcinoembryonic antigen; PSA = prostate-specific antigen; TSH = thyroid stimulating hormone (Thyrotropin); FT4 = free thyroxine; T3 = 3,5,3′-triiodothyronine; T4 = 3,5,3′,5′-tetraiodthyronine (Thyroxine).
[2] Percentage of healthy non-pregnant subjects.
[3] Percentage of healthy subjects.
[4] CEA (update): 0–100 ng/ml.

*Table 2(b). Specifications of comparative methods and instruments used in the evaluation.*

| Analyte | Method | Measuring range of test | Sample[1] volume | Incubation time | Detection limit | Instrument [Abbott][2] |
|---|---|---|---|---|---|---|
| AFP | MEIA | 0–350 ng/ml | 150 µl | 15 min | 0·4 ng/ml | AxSYM |
| CA 19-9 | MEIA | 0–500 IU/ml | 150 µl | 33 min | 2·0 IU/ml | AxSYM |
| CA 15-3 | MEIA | 0–250 U/ml | 150 µl | 45 min | < 0·2 U/ml | IMx |
| CA 125 | MEIA | 0–600 U/ml | 150 µl | 42 min | 0·45 U/ml | IMx |
| CEA | MEIA | 0–500 ng/ml | 150 µl | 30 min | 0·5 ng/ml | AxSYM |
| β-HCG | MEIA | 0–1000 mIU/ml | 150 µl | 9 min | 2·0 mIU/ml | AxSYM |
| PSA | MEIA | 0–100 ng/ml | 150 µl | 19 min | 0·1 ng/ml | AxSYM |
| TSH | MEIA | 0–100 mIU/l | 150 µl | 18 min | 0·08 mIU/l | AxSYM |
| FT4 | MEIA | 0–77 pmol/l | 150 µl | 18 min | 5·13 pmol/l | AsSYM |
| T3 | MEIA | 0–80 ng/ml | 150 µl | 26 min | 0·3 ng/ml | AxSYM |
| T4 | FPIA | 0–24 µg/dl | 150 µl | 10 min | 1·05 µg/dl | AxSYM |

[1] Including additional volume for postdilution and dead volume when Abbott sample cups were used.
[2] Abbott Laboratories, North Chicago, Illinois, USA.

cence polarization immunoassay (FPIA) and ICIA (Ion-capture immunoassay) [2, 3, 4].

The Abbot IMx System is an automated immunoassay analyser system using two different technologies, MEIA and FPIA. Specifications of the comparative methods are shown in table 2(*b*).

*Precision study*

Intra- and inter-assay precisions were determined using the BioRad Lyphochek Immunoassay Control Serum Level 1, 2 and 3 (BioRad, USA; Item No. C-37-5-IA, Lot No. 93000). This multicontrol is an artificial lyophilised control serum prepared from human serum. It was used for all thyroid hormones and all tumour markers, except for the tumour markers CA 15-3, CA 125 II, and CA 19-9, where the BIOREF RM16 Multimarker Control (BIOREF GmbH, Germany; Item and Lot No. 920161) was used and for CA 15-3 the BioRad Lyphochek Tumour Marker Control Level 1 and 2 (BioRad Item No. 580, Lot No. 64200) was used. All analytes in the precision study were checked with the lot specific ready-to-use test kit control and with a dedicated human serum pool, prepared, aliquoted and frozen at −20°C at the beginning of the second phase.

The precision acceptance criteria for tumour markers were taken from *Quality Control and Standardization of Tumour Marker Test* [6], and the criteria for TSH were taken from Jay *et al.* [7].

*Intra-assay imprecision*

For the intra-assay imprecision, the corresponding quality control sera and human serum pools were analysed in two independent runs with 20 replicates covering a time period of at least 14 days. The following analytes were checked: AFP, CA 15-3, CEA (update), PSA, TSH. FT4.

The lyophilized control sera were reconstituted freshly before each run. A human serum pool for each analyte was freshly prepared before each run. Mean, standard deviation and coefficients of variation (CVs) were calculated for each day.

*Inter-assay imprecision*

For the determination of between-series imprecision, the corresponding quality control sera and human serum pools were assayed in duplicates in up to 13 independent runs covering a time period of at least seven days for the following analytes: AFP, CEA, CEA (update), CA 15-3, PSA, TSH, FT4, and T3. Mean, standard deviation, and CVs were calculated by using all data obtained.

*Daily quality-control run*

To estimate the imprecision under real laboratory conditions, quality control results produced in August 1995 and October 1995 were analysed. This was done on the COBAS Core II and on the Abbott AxSYM using BioRad Lyphochek Immunoassay Control Serum levels 1, 2, and 3. The number of results per month ranged from 10 to 20.

*Accuracy*

The values obtained from the first three runs in the inter-assay imprecision analysis were compared with the assigned values of the different control materials (if available for the analyte) to estimate the accuracy of the COBAS Core EIAs.

*Dilution*

To compare automatic dilution with manual dilution, the accuracy and reproducibility of the automatic dilution feature of the COBAS Core II was estimated.

The PSA concentration in a human serum pool was determined for three dilution ratios (1:10, 1:50, 1:100) and in a single human sample with two dilution ratios (1:50, 1:100). The dilutions were performed using the automatic dilution feature of the COBAS Core II, and by manual dilution. The diluted samples were assayed in duplicate on two consecutive days. Both samples were diluted using the Cobas Diluent I.

In addition, the inter-assay imprecision of the CEA assay was determined using a human serum pool, the inter-assay imprecision of the AFP assay was determined using the Lyphocheck Immunoassay Control Serum Level 3, both automatically diluted 1:10 on the COBAS Core II.

*Method comparison*

A method comparison was performed using approximately 600 routine patient samples. TSH, T3, T4, AFP, CEA, and PSA were measured in parallel on COBAS Core II and Abbott AxSYM; CA 125 and CA 15-3 results were compared against the Abbott IMx. The comparison between the COBAS Core II and the Abbott AxSYM results was performed using Passing and Bablok's regression analysis [8, 9, 10]. Bland and Altmann's graphical presentation [11] was used to assess the degree of agreement between the two methods.

All sera were measured singly. Where there was a discrepancy the two methods were repeated if sufficient serum volume was available. Discrepant results were detected on the basis of the Bland and Altman analysis [11]. They were considered to be discrepant if they exceeded the 95% confidence belt for the difference of the two methods. Discrepant values which could not be repeated due to lack of serum were included in the calculation and were also listed in a separate table.

*Time study*

Direct operator times over a period of five days were calculated according to the guidelines developed by a joint working group of the Austrian, German, and Swiss Societies of Clinical Chemistry and for Laboratory Medicine [12].The time required to perform $N$ tests can be calculated by using the equation:

$$t_p = t_f + t_v \cdot N$$

where $t_p$ is the operator time; $t_f$ is the sum of fixed times—start-up time, preparation time for reagents, supply of consumables, waste disposal, preparation time for calibrators, controls and reagents, all maintenance, and shut-down procedures; $t_v$ is the sum of variable times (for $N = 1$ analysis)—all procedures dealing with preparation and handling of the samples, for example pipetting, diluting, and placing the samples on the instrument; $N$ is the number of the analyses performed.

The 'fixed' and 'variable' times were measured with the COBAS Core II and the Abbott AxSYM under the following laboratory conditions—use of barcoded primary tubes; manual order entry for patient samples; different tumour marker and endocrinology assays installed on both analysers.

### Results and discussion

*Imprecision*

The results of the intra- and inter-assay imprecision are summarized in table 3(*a*). Both the COBAS Core assays and the COBAS Core II system demonstrated good reproducibility overall.

*Intra-assay imprecision*

All thyroid assays were very precise, with intra-assay CVs ranging from 1·9 to 4·8%, verifying the assay specifications given by the manufacturer of ≤5·5% for TSH and ≤6·0% for FT4.

The intra-assay imprecision was between 1·8 and 4·9% for the tumour marker assays for all sample materials used. Only PSA gave a CV of 6·4% at a very low concentration of 0·6 ng/ml, far from the cut-off value of 4 ng/ml. All CVs, except for the low level serum pool for PSA, were within the limits (<5%) given in *Quality Control and Standardization of Tumour Marker Tests* [6].

*Inter-assay imprecision*

CVs for the TSH assay ranged from 2·1% to 4·2%. No value exceeded the acceptance limits of 10–15% set by the American Thyroid Association [7] and the specifications of Roche Diagnostics of ≤6·5%.

The imprecision for FT4 was less than 5·7% for the low and medium conrol sera and for the human serum pool. The CV for the high control sera was 8·5% and therefore outside the manufacturer's specification of ≤8%. However, the concentration level of these samples (72·3 pmol/l) was outside the clinical decision range.

The CVs for T3 ranged from 2·1 to 7·7%, and were therefore all within the manufacturer's specification (≤8%).

In addition, the imprecision for the selected thyroid panel showed good correspondence to results found in a recently published thyroid study [13], although a better imprecision for T3 in the high measuring range was obtained.

Inter-assay CVs for all tumour markers tested ranged from 2·0 to 7·8%, therefore they met the specifications for the inter-assay imprecision of <5–8%, suggested in *Quality Control and Standardization of Tumour Marker Tests* [6].

The imprecision for the COBAS Core CEA assay was close to the upper limit of the specification claimed by the manufacturer (≤8%). An improved version of the COBAS CEA EIA, which is currently in development, was tested and found to give a significantly better precision with a median CV of 5·7%.

The measured values for the COBAS Core PSA assay agreed with the data published by Schambeck *et al.* [14].

The inter-assay imprecision for the CA 15-3 EIA assay (CV less than 5%) was superior to results reported in an evaluation of three CA 25-3 assays [15].

*Daily quality-control run*

Results of the daily control runs are presented in figure 1. Similar CVs between COBAS Core II and Abbott AxSYM were observed for the following assays: T3 (except Lyphochek Level 3 where AxSYM showed better results), β-hCG, CEA, CA 19-9 and PSA (but there was a larger span of CVs for AxSYM). The COBAS Core II showed better CVs for TSH and AFP, whereas the T4 assay on the Abbott AxSYM was more precise.

*Table 3(a). Intra-assay and inter-assay imprecision of the COBAS Core II. Mean values and coefficients of variation.*

| | | | Intra-assay | | | | Inter-assay | | |
|---|---|---|---|---|---|---|---|---|---|
| | | | Mean | | CV% | | | | |
| Analyte[1] | Control material | Assigned value | Day 1 | Day 2 | Day 1 | Day 2 | Mean | CV% | *N* |
| AFP (ng/ml) | Lyphochek I | 25·0 | 26·9 | 27·6 | 3·1 | 2·7 | 25·4 | 3·1 | 26 |
| | Lyphochek II | 112·0 | 122·4 | 127·5 | 2·8 | 3·0 | 118·2 | 7·2 | 26 |
| | Kit control | 31·3 | 31·4 | 32·4 | 3·3 | 2·6 | 31·2 | 3·2 | 26 |
| | Human serum pool | — | 29·1 | 1·4[4] | 3·2 | 4·9 | 8·5 | 2·5 | 26 |
| CA 15-3 (U/ml) | Lyphochek I | — | — | — | — | — | 13·7 | 5·0 | 26 |
| | Lypho. Tumor Ctrl. 1 | 11·0 | — | 12·5 | — | 2·5 | — | — | — |
| | Lypho. Tumor Ctrl. 2 | 32·0 | — | 32·9 | — | 3·2 | — | — | — |
| | Bioref RM 16 low | 19·0 | 18·9 | 18·7 | 2·6 | 3·1 | 19·5 | 4·3 | 26 |
| | Bioref RM 16 high | 67·0 | 63·5 | 63·8 | 2·9 | 3·1 | 66·7 | 4·6 | 26 |
| | Kit control | 31·0 | 30·4 | 30·8 | 2·5 | 2·3 | 31·1 | 4·4 | 26 |
| | Human serum pool | — | — | — | — | — | 77·6 | 3·6 | 26 |
| CEA (ng/ml) | Lyphochek I | 2·8 | — | — | — | — | 2·8 | 6·7 | 26 |
| | Lyphochek II | 13·1 | — | — | — | — | 13·5 | 7·8 | 26 |
| | Lyphochek III | 29·6 | — | — | — | — | 29·5 | 6·0 | 26 |
| | Kit control | 7·5 | — | — | — | — | 7·5 | 6·5 | 26 |
| | Human serum pool | — | — | — | — | — | 13·0 | 5·2 | 24[2] |
| CEA (update) (ng/ml) | Lyphochek I | (2·8)[3] | 2·7 | 2·6 | 3·8 | 4·1 | 2·5 | 5·8 | 26 |
| | Lyphochek II | (13·1) | 11·3 | 11·7 | 2·7 | 2·3 | 11·8 | 5·7 | 24 |
| | Lyphochek III | (29·6) | 24·6 | 26·5 | 3·8 | 3·6 | 26·6 | 3·9 | 26 |
| | Human serum pool A | — | 2·8 | 2·6 | 4·3 | 3·7 | 5·5 | 5·0 | 22[2] |
| | Human serum pool B | — | 19·6 | 18·7 | 2·5 | 2·2 | 38·7 | 5·7 | 22[2] |
| PSA (ng/ml) | Lyphochek I | 2·6 | 2·6 | 2·7 | 3·3 | 3·1 | 2·6 | 4·2 | 26 |
| | Lyphochek II | 8·6 | 10·0 | 9·9 | 1·8 | 2·8 | 9·9 | 3·2 | 26 |
| | Lyphochek III | 79·8 | 95·4 | 92·4 | 2·2 | 3·4 | 91·7 | 2·0 | 26 |
| | Kit control | 10·9 | 11·0 | 11·0 | 2·3 | 2·3 | 10·8 | 2·4 | 26 |
| | Human serum pool | — | 0·6 | 0·6 | 6·4 | 6·1 | 77·5 | 2·9 | 26 |
| TSH (mIU/l) | Lyphochek I | 0·97 | 0·94 | 0·94 | 3·3 | 4·5 | 0·89 | 4·2 | 26 |
| | Lyphochek II | 6·4 | 6·6 | 6·6 | 2·2 | 2·8 | 6·7 | 2·9 | 26 |
| | Lyphochek III | 22·5 | 24·2 | 23·3 | 2·4 | 2·0 | 23·7 | 2·2 | 26 |
| | Kit control | 2·4 | 2·5 | 2·4 | 2·1 | 1·6 | 2·5 | 2·1 | 26 |
| | Human serum pool | — | 1·1 | 1·1 | 1·9 | 2·1 | 2·1 | 2·2 | 26 |
| FT4 (pmol/l) | Lyphochek I | 6·3 | 6·7 | 6·4 | 2·5 | 2·6 | 6·6 | 4·5 | 26 |
| | Lyphochek II | 17·9 | 20·8 | 18·8 | 3·1 | 2·7 | 19·8 | 3·0 | 26 |
| | Lyphochek III | 83·2 | 81·2 | 73·2 | 3·2 | 4·8 | 72·3 | 8·5 | 26 |
| | Kit control | 12·0 | 11·7 | 10·1 | 3·8 | 4·7 | 10·9 | 7·1 | 26 |
| | Human serum pool | — | 16·6 | 14·6 | 2·1 | 3·5 | 15·6 | 5·7 | 26 |
| T3 (ng/ml) | Lyphochek I | 0·9 | — | — | — | — | 0·9 | 5·4 | 26 |
| | Lyphochek II | 2·0 | — | — | — | — | 2·0 | 3·2 | 26 |
| | Lyphochek III | 4·2 | — | — | — | — | 3·3 | 6·1 | 26 |
| | Calibrator 3c | 1·0 | — | — | — | — | 1·1 | 7·7 | 26 |
| | Human serum pool | — | — | — | — | — | 1·8 | 2·1 | 26 |

[1] Full calibration: CA 15-3, CEA (update), PSA, FT4; Recalibration: AFP, CEA, TSH, T3.
[2] Insufficient serum available.
[3] Values in brackets: assigned values not yet specified for CEA (update).
[4] Different human serum pool on day 2.

### *Accuracy*

The results of the comparison of mean values with assigned values of control samples are listed in table 3(*b*). All results were within the acceptable range defined for COBAS Core EIAs.

The mean values obtained with the CEA (update) assay for the Lyphochek Immunoassay Control Serum Levels 1, 2, and 3 were 10% below the assigned value. Since the target value for this new assay still has to be established, the assigned value for the existing CEA assay was used for the evaluation of results.

### *Automatic versus manual dilution*

The dilution performed by COBAS Core II was more precise than the manual dilutions for all dilution ratios investigated. This is obvious from both the scatter of the replicate values and the mean values given in figure 2(*a*) and 2(*b*), and table 4. However, a single outlier was

*Table 3(b). Accuracy with control sera: recovery range.*

| Analyte | Control material | Assigned value | Mean value | Accuracy recovery range %min | %max |
|---|---|---|---|---|---|
| AFP | Lyphochek I | 25·0 | 25·8 | 100 | 107 |
| (ng/ml) | Lyphochek II | 112·0 | 120·4 | 104 | 110 |
| | Lyphochek III | 248·0 | 254·9 | 95 | 104 |
| | Kit Control | 31·3 | 31·1 | 94 | 103 |
| Ca 15-3 | Bioref RM 16 low | 19·0 | 20·0 | 102 | 111 |
| (U/ml) | Bioref RM 16 high | 67·0 | 67·7 | 99 | 107 |
| | Kit Control | 31·0 | 32·3 | 100 | 111 |
| CEA | Lyphochek I | 2·8 | 2·9 | 93 | 107 |
| (ng/ml) | Lyphochek II | 13·1 | 14·1 | 92 | 116 |
| | Lyphochek III | 29·6 | 29·1 | 90 | 103 |
| | Kit Control | 7·5 | 7·4 | 92 | 107 |
| CEA | Lyphochek I | (2·8)[1] | 2·5 | (84 | 99) |
| (update) | Lyphochek II | (13·1) | 11·8 | (83 | 93) |
| (ng/ml) | Lyphochek III | (29·6) | 25·6 | (84 | 90) |
| PSA | Lyphochek I | 2·6 | 2·6 | 95 | 109 |
| (ng/ml) | Lyphochek II | 8·6 | 10·0 | 112 | 119 |
| | Lyphochek III | 79·8 | 91·5 | 97 | 117 |
| | Kit Control | 10·9 | 10·9 | 97 | 101 |
| TSH | Lyphochek I | 0·97 | 0·90 | 85 | 93 |
| (mIU/l) | Lyphochek II | 6·4 | 6·6 | 101 | 108 |
| | Lyphochek III | 22·5 | 23·6 | 100 | 108 |
| | Kit Control | 2·4 | 2·4 | 99 | 105 |
| FT4 | Lyphochek I | 6·3 | 6·5 | 100 | 110 |
| (pmol/l) | Lyphochek II | 17·9 | 19·7 | 107 | 113 |
| | Lyphochek III | 83·2 | 68·9 | 77 | 90 |
| | Kit Control | 12·0 | 11·1 | 83 | 97 |
| T3 | Lypochek I | 0·9 | 0·9 | 93 | 104 |
| (ng/ml) | Lyphochek II | 2·0 | 2·0 | 99 | 111 |
| | Lyphochek III | 4·2 | 3·2 | 73 | 81 |

[1] Values in brackets: assigned values not yet specified for CEA (update).

observed on the second day with the automatic dilution 1:100, for which no explanation could be given.

The inter-assay CV for a human serum pool (CEA concentration = 96 ng/ml) and for a Lyphochek Control Serum (AFP concentration = 252 ng/ml) both automatically diluted to 1:10 by the COBAS Core II system. An inter-run variation of 7·6% was observed for CEA, a CV of 3·2% was observed for AFP. This is within the range of CVs found with undiluted samples for the COBAS Core CEA assay (see table 3[*a*]).

### *Method Comparison*

Table 5 lists the numerical data obtained in the method comparison. Diagrams obtained in the method comparison study are shown in figure 3. Method comparisons for TSH and T4 yielded Spearman coefficients of correlation ($r_s$) of 0·97 and 0·917, respectively. The slopes of the regression equation were close to unity, with the values for TSH and T4 in the COBAS Core assay differing from those in the Abbott AxSYM by 8% and 12%, respectively. The data for T3 showed wider spreading around the regression line as did TSH and T4 ($r_s = 0{\cdot}784$).

For nine patient samples, significantly different results were found for T3 and T4. These differences were confirmed by a rerun of the same sample on both analysers, as well as determination of T3 and T4 in up to four different samples from the same patients. The reasons for the discrepant T3 and T4 results could be due to human anti-mouse IgG antibodies (HAMA, heterophilic antibodies) being formed in patients who have received mouse immunoglobulins as part of diagnostic immunoscintigraphy or immunotherapy. These can react with reagent antibodies, leading to false-positive results. These heterophilic anti-IgG antibodies can also occur in patients who have had fresh-cell therapy. So, samples with very high values were excluded as some of the cancer patients had received immunotherapy; the high T3 and T4 values were probably due to these heterophilic antibodies.

In addition, some drugs cause increased protein binding, leading to increased serum T3 and T4 levels. Therefore, T3 and T4 tests may be abnormal. Different test methods may be affected differently by these conditions. Data on the use of drugs for patient treatment were not available. Some values measured in severely ill patients were excluded from the correlation study, because it is known that critical illness can have significant effects on thyroid function tests.

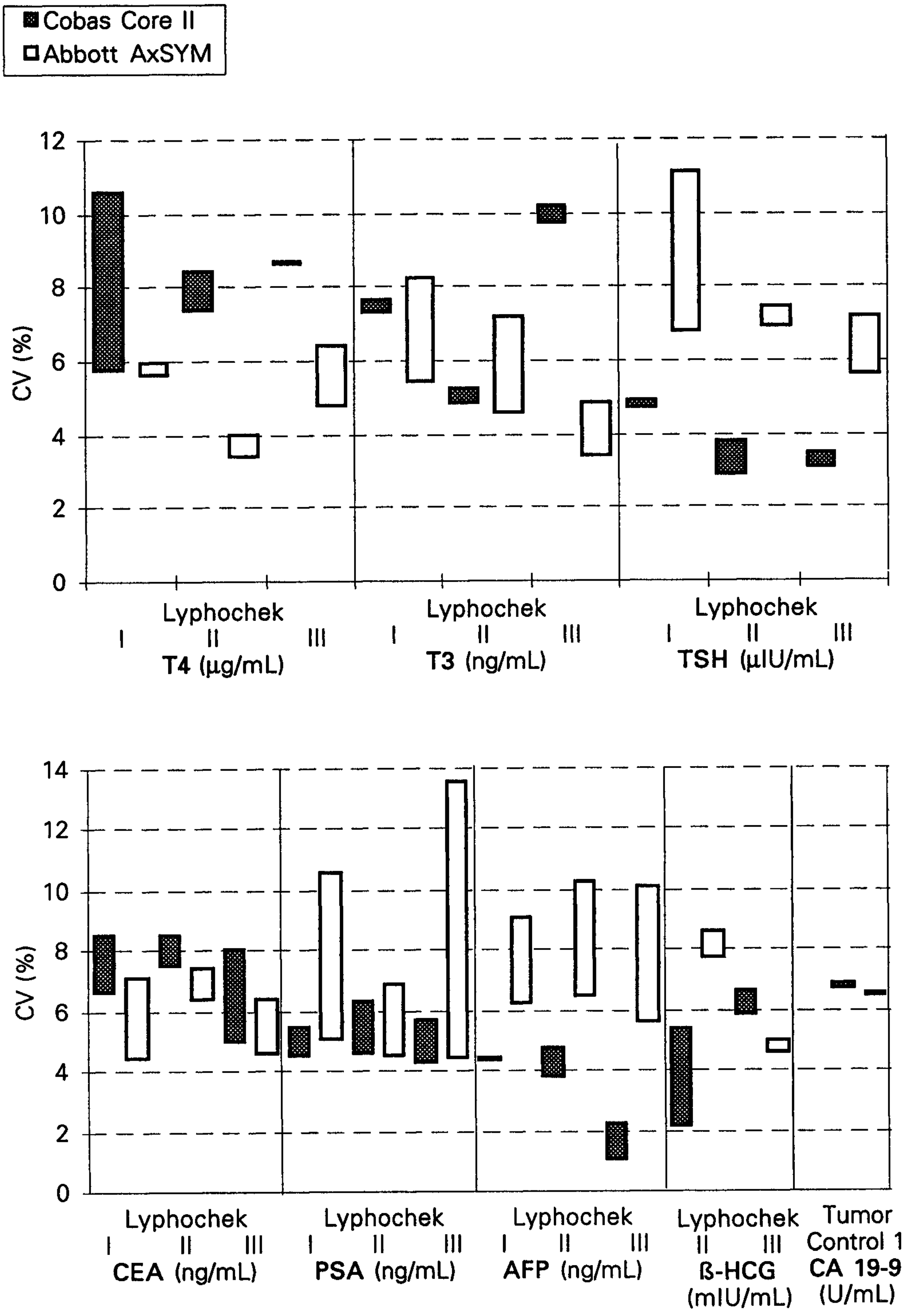

*Figure 1. Precision results of the daily quality control (bars represent maximum and minimum variation in the daily quality control as measured in August and October).*

Very good agreement was observed when comparing the COBAS Core AFP assay with the AFP assay on Abbott AxSYM.

Good coefficients of correlation (all above 0·98) for CA 125 and CA 15-3 were found when comparing the COBAS Core assays with the routine method on the Abbott IMx System. One discrepant result was found in the method comparison CA 125 (COBAS Core II: 3010 U/ml, Abbott IMX 1295 U/ml). A possible explanation could be the use of different catcher antibodies and therefore a slightly different specificity. In addition, the difference of the results is clinically not relevant.

A slope of 1·28 was found for the COBAS Core CEA assay. It is reported that certain tumour marker values, particularly CEA, are highly dependent upon the method used [16]. The different antibodies used in the

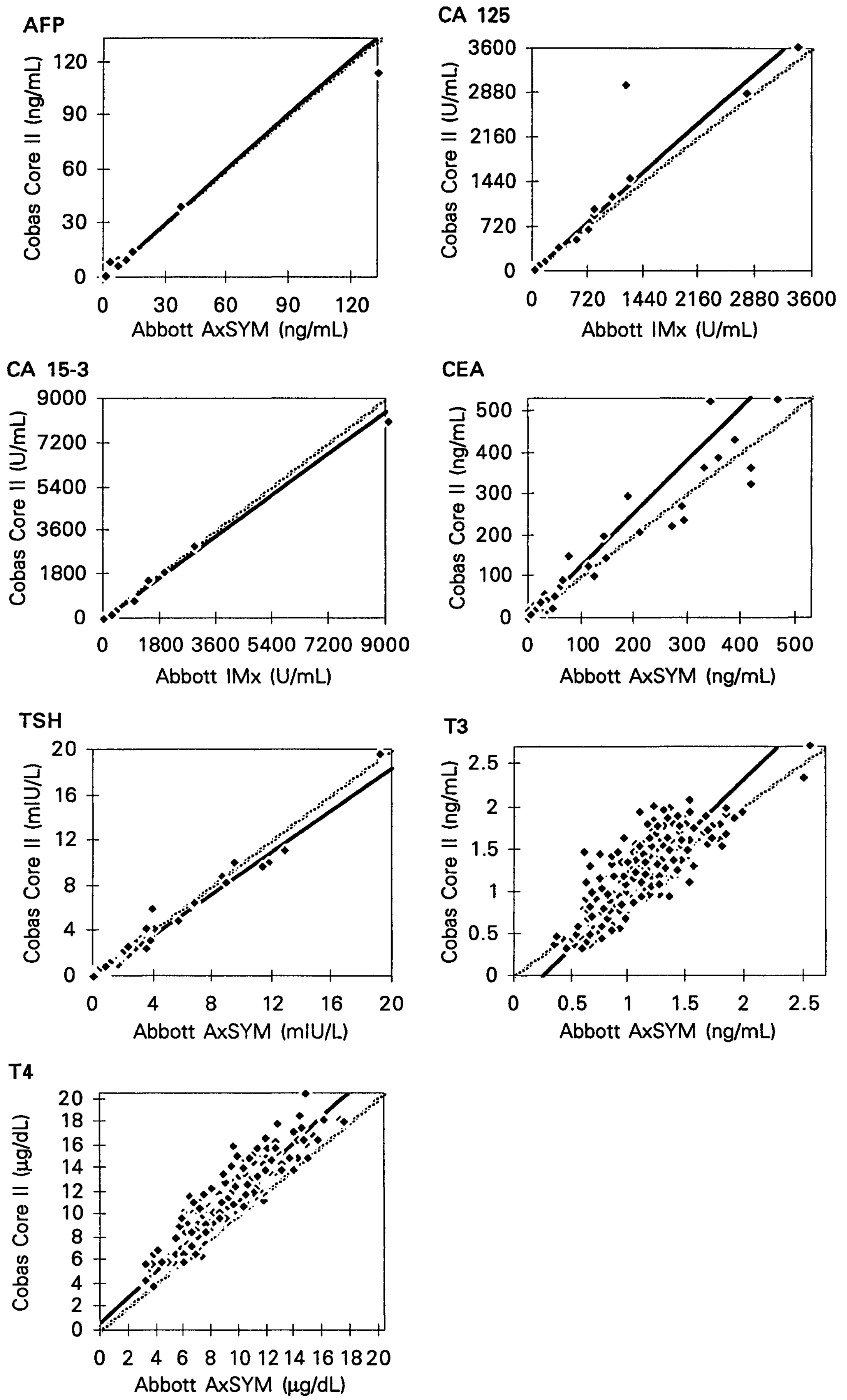

*Figure 2. Diagrams obtained in the method comparison study.*

*Table 4. Comparison of automatic versus manual dilution for PSA in ng/ml.*

| | | Automatic dilution | | Manual dilution | |
|---|---|---|---|---|---|
| Dilution | | Pool | Specimen | Pool | Specimen |
| 1:10 | Median | 389 | — | 379 | — |
| | Min | 387 | — | 361 | — |
| | Max | 406 | — | 390 | — |
| | Range | 19 | — | 29 | — |
| 1:50 | Median | 396 | 1895 | 430 | 1878 |
| | Min | 376 | 1797 | 391 | 1756 |
| | Max | 411 | 1919 | 452 | 2025 |
| | Range | 35 | 122 | 61 | 270 |
| 1:100 | Median | 363 | 1845 | 408 | 1940 |
| | Min | 352 | 1673 | 391 | 1897 |
| | Max | 374 | 1870 | 431 | 1971 |
| | Range | 22 | 197 | 40 | 74 |

assays may have small differences in their affinity for CEA, leading to slightly different values between methods.

PSA results ranging from 0 ng/ml to 1040 ng/ml were found to be 42% higher in the COBAS Core PSA assay than in the Abbott PSA assay. However, when interpreting this finding, it is important to consider that the sample number was rather small ($N = 44$) and was not evenly distributed over the whole analytical range; and the COBAS Core PSA EIA is standardized against the Stanford standard with a PSA:ACT complex to free PSA ratio of 9:1. This complex/free PSA ratio cannot be assured in patients in later stages of cancer [17]. PSA concentrations of above 20 ng/ml were observed in 12 patient samples.

### *Time study*

Start-up time, supply of consumables, waste disposal, preparation time for calibrators, controls and reagents, and all maintenance and shut-down procedures were measured for 'fixed' times. The 'variable' times included all procedures dealing with preparation and handling of the samples.

For COBAS Core II a fixed time of 35 min was determined, and the variable time per test was 3·5 s. For comparison, the fixed time determined with the ACS: 180 in a multicentre evaluation [18] was 12·2 min, and the variable time was 12 s. In another time study performed with the Du Pont Dimension 380 [19] analyser, the fixed time was 26 min, and the mean value of the variable times was 2·5 s.

In order to compare these data with the times determined on the Abbott AxSYM, some COBAS Core II data were normalized to the true laboratory environment: the analyser is always in the standby mode (i.e. never switched off), equipped with an automatic water supply unit and connected to a laboratory information system. Under these circumstances, fixed times of 29 min were obtained for both analysers, whereas the variable time per test on COBAS Core II was slightly lower (1·1 s) than on Abbott AxSYM (1·9 s).

The short variable time on COBAS Core II is due to the automatic dilution feature, thus reducing sample preparation time and operator interaction to a minimum and therefore compensating for the time saving because of less post-diluted samples on Abbott AxSYM (higher measuring range of Abbott CEA and $\beta$-hCG assay).

The COBAS Core II requires about 10 min for daily maintenance and about 30 min for the weekly maintenance, whereas the Abbott AxSYM requires about 10 min for the daily, and about 40 min for the weekly maintenance.

Taking total processing times into account only, the Abbott AxSYM™ was faster than COBAS Core II (by an average of 44 min per day). However, only small batches of 12–26 samples per day were analysed in this time study. Therefore the shorter incubation time for some Abbott assays has more effect on the total processing time than does the shorter work cycle time of the COBAS Core II.

### *Routine simulation*

The ease of use and flexibility of the COBAS Core II analyser was assessed by running the system in a routine environment in parallel with the main routine analyser. In total, up to 26 samples per day, which is equal to 105 tests per day, were processed. The following assays were

*Table 5. Method comparison studies in human sera with COBAS Core II ($y$) and comparative methods ($x$). Regression analyses ($y = a + bx$) were performed according to Passing and Bablok, see text.*

| Analyte | Reference instrument | Regression equation: Passing–Bablok | Correlation: coefficient Spearman ($r_s$) | Sample size ($N$) | Minimal value | Maximal value |
|---|---|---|---|---|---|---|
| AFP | Abbott AxSYM | $y = 1{\cdot}01\,x - 0{\cdot}08$ | 0·932 | 30 | 1·33 ng/ml | 114 ng/ml |
| CA 125 | Abbott IMx | $y = 1{\cdot}11\,x + 5{\cdot}23$ | 0·981 | 24 | 6·4 U/ml | 3620 U/ml |
| CA 15-3 | Abbott IMx | $y = 0{\cdot}94\,x - 0{\cdot}22$ | 0·981 | 93 | 5·49 U/ml | 8080 U/ml |
| CEA | Abbott AxSYM | $y = 1{\cdot}28\,x + 0{\cdot}11$ | 0·942 | 174 | 0·4 ng/ml | 530 ng/ml |
| TSH | Abbott AxSYM | $y = 0{\cdot}92\,x + 0{\cdot}03$ | 0·970 | 378 | 0·0 mIU/l | 19·6 mIU/l |
| T3 | Abbott AxSYM | $y = 1{\cdot}29\,x - 0{\cdot}28$ | 0·784 | 290 | 0·34 ng/ml | 2·72 ng/ml |
| T4 | Abbott AxSYM | $y = 1{\cdot}12\,x + 0{\cdot}69$ | 0·917 | 295 | 3·76 µg/dl | 20·432 µg/dl |

used on both the COBAS Core II and the Abbott AxSYM: TSH, T4, T3, AFP, β-hCG, CA 19-9, CEA, and PSA. Barcoded samples were used on both analysers and, in addition, the COBAS Core II was working with barcoded control sera. The COBAS Core was not connected to the laboratory information system.

### Conclusions

Overall experience with the COBAS Core II was very positive. It is a well designed immunoassay system with some strong features:

(1) The software is user friendly, easy to learn and with a good screen design and presentation.
(2) The automatic pre- and postdilution is very flexible, exhibiting superior performance compared to manual dilution.
(3) With the colour coding concept of the software and the use of LED indicators for the continuous loading of the sample and reagents, the operator always has a good overview of the instrument status.
(4) The use of a factory-stored calibration curve saves time and reduces costs: for recalibration just one calibrator is required. In addition, calibration intervals for each test can be entered: when the defined interval for the calibration has been reached, the test concerned is highlighted.

### *Areas for improvement*

Improvements should be made in the following areas:

(*a*) The measuring range of the CEA and β-hCG assay should be extended for samples exceeding the existing measuring range (patients with malignant tumours).
(*b*) The quality control software should include a graphical display and validation criteria.
(*c*) An emergency stop feature should be added to allow the operator to interrupt the processing of samples.

### *Problems occurring during the evaluation*

In general, the COBAS Core II was very reliable, with only some minor instrument-related problems encountered during the evaluation. It should be noted that the instrument tested was from the first pilot production. On one occasion, a needle breakage occurred and the analyser did not stop automatically (Roche Diagnostics are correcting this problem).

In summary, COBAS Core II demonstrated good analytical performance and good practicability in routine use, especially for samples requiring a post-dilution step. Along with the broad consolidated test menu of 45 tests, this analyser proved to be extremely efficient.

### *Manufacturers' comments*

Some small improvements have now been made to the instrument to solve the problems which occurred during the evaluation. As part of a continuous development programme, updated versions of CEA and β-HCG with an extended measuring range will shortly be launched.

### Acknowledgement

The authors wish to thank Roche Diagnostics for placing the instrument and the necessary reagents at their disposal.

### References

1. Okuda, K., *Journal of Clinical Chemistry and Clinical Biochemistry*, **18** (1980), 947.

2. Costongs, G., van Oers, R., Leerkens, B., Hermans, W. and Janson, P., *European Journal of Clinical Chemistry and Clinical Biochemistry*, **33** (1995), 105.

3. Smith, J., Osikowicz, G. *et al.*, *Clinical Chemistry*, **39** (1993), 2063.

4. Prevosto, J. M., Renard, C., Rault, J. P., Cheminel, V., Caire-Maurisier, A. M. Grafmeyer, D. and Chaulet, J. F., OPTION/BIO supplément au no 141 (1995), ISB No 35.

5. Neuwald, C., Bauer, K., Kittl, E. M., Sitte, S. and Fischer, G., *Laboratory Medicine*, **14** (1990), 73.

6. Klapdor, R., *Tumordiagn. u. Ther.*, **13** (1992), XIX–XXII and van Dalen. A., *Tumor Biology.*, **14** (1993), 131.

7. Hay, I. D. *et al.*, *Clinical Chemistry*, **37** (1991), 2002.

8. Passing, H. and Bablok, W., *Journal of Clinical Chemistry and Clinical Biochemistry*, **21** (1983), 709.

9. Passing, H. and Bablok, W., *Journal of Clinical Chemistry and Clinical Biochemistry*, **22** (1984), 431.

10. Bablok, W., Passing, H., Bender, R. and Schneider, B., *Journal of Clinical Chemistry and Clinical Biochemistry* **26** (1988), 783.

11. Bland, J. M. and Altmann, D. G. *The Lancet*, **1** (1986), 307.

12. Haeckel, R., Bayer, M., Fang-Kirchner, S., Fischer, G., Gibitz, H. J., Hinsch, W. and Weidemann, G., In *Evaluation Methods in Laboratory Medicine* (VCH Verlag, Weinheim, 1993).

13. Michotey, M. *et al.*, *European Journal of Clinical Chemistry and Clinical Biochemistry*, **33** (1995), 609.

14. Schambeck, M. *et al.*, *European Journal of Clinical Chemistry and Clinical Biochemistry*, **33** (1995), 541.

15. Ammon, A. and Siehl, S., *Tumordiagn. u. Ther.*, **16** (1995), 232.

16. Fateh-Moghadam, A. and Stieber, P., In *Sensible Use of Tumour markers* (Editiones Roche, Basel, 1993).

17. Wood, W. G., van der Sloot, E. and Böhle, A., *European Journal of Clinical Chemistry and Clinical Biochemistry*, **29** (1991), 787–794.

